# A Quasi-Experimental Evaluation of a Nutrition Behavior Change Intervention Delivered Through Women's Self-Help Groups in Rural India: Impacts on Maternal and Young Child Diets, Anthropometry, and Intermediate Outcomes

**DOI:** 10.1093/cdn/nzac079

**Published:** 2022-04-11

**Authors:** Samuel Scott, Shivani Gupta, Purnima Menon, Kalyani Raghunathan, Giang Thai, Agnes Quisumbing, Vandana Prasad, Aditi Hegde, Avijit Choudhury, Madhu Khetan, Carly Nichols, Neha Kumar

**Affiliations:** Poverty Health and Nutrition Division, International Food Policy Research Institute, New Delhi, India; Poverty Health and Nutrition Division, International Food Policy Research Institute, New Delhi, India; Poverty Health and Nutrition Division, International Food Policy Research Institute, New Delhi, India; Poverty Health and Nutrition Division, International Food Policy Research Institute, New Delhi, India; Poverty Health and Nutrition Division, International Food Policy Research Institute, Washington, DC, USA; Poverty Health and Nutrition Division, International Food Policy Research Institute, Washington, DC, USA; Public Health Resource Society (PHRS), New Delhi, India; Public Health Resource Society (PHRS), New Delhi, India; Professional Assistance for Development Action (PRADAN), New Delhi, India; Professional Assistance for Development Action (PRADAN), New Delhi, India; Geographical and Sustainability Studies Department, University of Iowa, Iowa City, IA, USA; Poverty Health and Nutrition Division, International Food Policy Research Institute, Washington, DC, USA

**Keywords:** infant and young child feeding, anthropometry, India, women's self-help groups, behavior change communication

## Abstract

**Background:**

Women's self-help groups (SHGs) have become one of the largest institutional platforms serving the poor. Nutrition behavior change communication (BCC) interventions delivered through SHGs can improve maternal and child nutrition outcomes.

**Objectives:**

The objective was to understand the effects of a nutrition BCC intervention delivered through SHGs in rural India on intermediate outcomes and nutrition outcomes.

**Methods:**

We compared 16 matched blocks where communities were supported to form SHGs and improve livelihoods; 8 blocks received a 3-y nutrition intensive (NI) intervention with nutrition BCC, and agriculture- and rights-based information, facilitated by a trained female volunteer; another 8 blocks received standard activities (STD) to support savings/livelihoods. Repeated cross-sectional surveys of mother-child pairs were conducted in 2017–2018 (*n* = 1609 pairs) and 2019–2020 (*n* = 1841 pairs). We matched treatment groups over time and applied difference-in-difference regression models to estimate impacts on intermediate outcomes (knowledge, income, agriculture/livelihoods, rights, empowerment) and nutrition outcomes (child feeding, woman's diet, woman and child anthropometry). Analyses were repeated on households with ≥1 SHG member.

**Results:**

Forty percent of women were SHG members and 50% were from households with ≥1 SHG member. Only 10% of women in NI blocks had heard of intervention content at endline. Knowledge improved in both NI and STD groups. There was a positive NI impact on knowledge of timely introduction of animal-sourced foods to children (*P* < 0.05) but not on other intermediate outcomes. No impacts were observed for anthropometry or diet indicators except child animal-source food consumption (*P* < 0.01). In households with ≥1 SHG member, there was a positive NI impact on child unhealthy food consumption (*P* < 0.05).

**Conclusions:**

Limited impacts could be due to limited exposure or skills of volunteers, and a concurrent national nutrition campaign. Our findings add to a growing literature on SHG-based BCC interventions and the conditions necessary for their success.

## Teaser

Community-level impacts of a volunteer-led behavior change intervention delivered through women's self-help groups in 5 Indian states on nutrition outcomes in mothers and their young children were investigated.

## Introduction

Progress toward reducing maternal and child undernutrition in low- and middle-income countries is uneven, with most countries not on track to meeting nutrition targets outlined by the World Health Assembly and Sustainable Development Goals (1). Given its large population and high prevalence of undernutrition, India accounts for a large share of the global burden. Initial results from India's fifth National Family Health Survey, based on data collected in 2019–2021, have suggested a stagnant or worsening nutrition situation since 2015–2016 in many states; these results have raised questions about the ability of the country's nutrition and social protection programs to buffer against slowing economic growth and increasing inequalities (2). With the COVID-19 crisis deepening the undernutrition problem further (3), understanding effective solutions is an urgent priority.

New evidence acknowledges the diversity of drivers of undernutrition and the need for direct and indirect multisectoral community-based approaches focusing on women of reproductive age, in particular efforts that directly reach and target women around the health, economic, and social causes of malnutrition (4). In India, the combined efforts of nongovernmental organizations and the government's National Rural Livelihood Mission (NRLM) and National Health Mission have led to a massive scaling up of women's groups in the last 2 decades. In April 2022, >82 million households included members of NRLM self-help groups (SHGs) (5). Given the extensive reach of women's groups in India, particularly into rural populations that health system interventions have difficulty reaching and where malnutrition prevails, the SHG platform has emerged as a potential platform for delivering nutrition-focused behavior change communication (BCC) interventions (6).

Several evaluations in India have examined impacts of group-based nutrition BCC interventions on maternal and child diet and health outcomes. The findings from these studies have been recently summarized (7, 8). One set of interventions implemented by Ekjut, a nonprofit organization in India (http://www.ekjutindia.org/), involve extensively trained, paid female workers facilitating a participatory learning and action (PLA) cycle of SHG meetings and conducting home visits to provide health counseling to pregnant women and young mothers (9–12). Cluster randomized controlled trials (RCTs) have shown significant impacts of Ekjut's interventions in 2 states of India, Jharkhand and Odisha, on women's dietary diversity, hygiene behavior, child feeding practices, child anthropometry, and child mortality while being cost-effective (11–13). The Ekjut trials, along with evidence from Bangladesh, Nepal, and Malawi (14), led to a WHO recommendation to include PLA through women's groups to improve maternal and newborn health (15). Most recently, the addition of nutrition and agriculture videos to the PLA meetings has been reported to benefit diet quality but not anthropometric outcomes (13). An additional example comes from the JEEViKA Multisectoral Convergence (JEEViKA-MC) pilot in Bihar, also a cluster RCT involving nutrition BCC through SHGs. The JEEViKA-MC pilot found no impacts on anthropometry in mothers of young children or their children but small positive impacts on dietary diversity, and was delivered with per member per year costs comparable to those of the Ekjut interventions (16). An important difference was that the JEEViKA-MC intervention did not follow a PLA cycle and was delivered through NRLM SHGs, thus the study was able to assess impacts through an already-scaled platform. Lastly, a quasi-experimental evaluation of a health BCC intervention delivered by female volunteers through microfinance-based SHGs in Uttar Pradesh found impacts on maternal and newborn health practices, especially in the most marginalized groups, but did not report on diets or anthropometry (17). Taken together, the evidence suggests that SHG-based interventions can improve diets but are unlikely to improve anthropometry.

Several research gaps remain. First, there is a consensus among nutrition implementation science researchers that understanding why programs were successful or not requires measurement beyond nutrition outcomes (18). Our review of women's group–based nutrition interventions in South Asia found that measurement of the intermediate outcomes along multiple impact pathways is often overlooked (19). Second, additional evidence is needed on the effectiveness of models involving unincentivized female volunteers without any accompanying in-kind or cash transfer to beneficiaries, that is, provision of information only (7), given the high cost of financing a national effort to deliver and sustain delivery of nutrition BCC to the millions of Indian women who participate in SHGs. Third, evidence on the marginal benefit of adding nutrition BCC to agriculture-focused SHGs is needed because most rural Indians depend on agriculture for their livelihoods, and agriculture's importance to nutrition is well recognized (20, 21). However, most evaluations of agriculture-nutrition interventions, such as those of homestead food production programs, examine the impact of bundled programs, not the impact of a BCC intervention layered onto an agriculture platform. Finally, because SHG members are supposed to share learnings with the wider community of nonmembers, experts have recommended that studies of group-based health interventions should estimate population-level effects and not only focus on group members (7). This is particularly important for our study because the target of many SHG-based health interventions is women entering pregnancy or with young children, who might not have joined an SHG yet; on average, women in government SHGs in India are 38 y old and could already have completed childbearing (22).

Our study aimed to answer the following research questions: *1*) What impact did an information-only nutrition BCC intervention delivered to women members through SHGs in 5 Indian states have on maternal and young child diet and anthropometry at the population level and, additionally, among women and children living in households with an SHG member? and *2*) To what extent did this intervention benefit intermediate outcomes related to income generation, agricultural livelihoods, health- and nutrition-related behavior change, rights, and women's empowerment?

## Methods

### Study setting

The study was conducted in rural villages in 16 blocks across 8 districts in 5 states where an Indian nongovernmental organization called Professional Assistance for Development Action (PRADAN) has worked for 2 decades: West Bengal, Jharkhand, Orissa, Madhya Pradesh, and Chhattisgarh. PRADAN focuses on improving women's livelihoods by forming and strengthening women's savings and credit SHGs and training members in agriculture, livestock, and natural resource management. As of March 2020, PRADAN had established 65,000 SHGs reaching 862,000 households across 7 states.

### Intervention description

**FIGURE 1:**
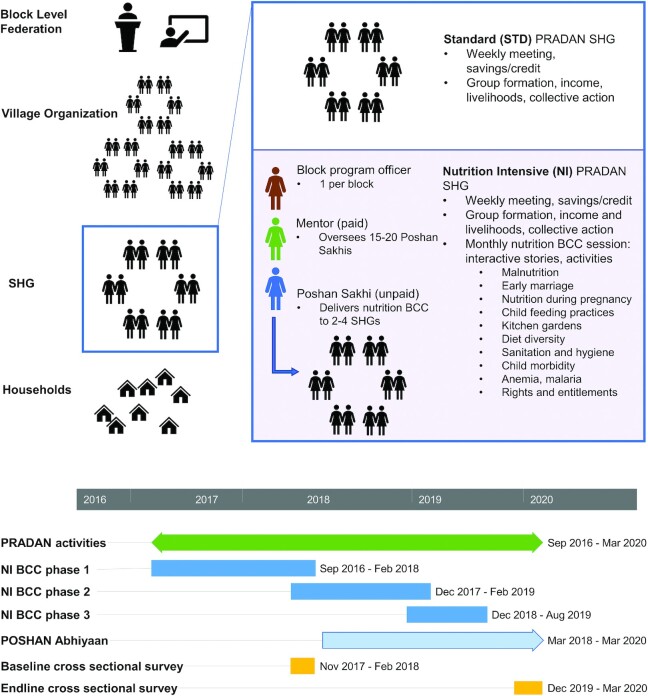
Intervention description and timing. PRADAN's standard set of activities focused on SHG formation and agricultural livelihoods. The BCC interventions were added on in the 8 NI study blocks and were delivered at PRADAN SHG meetings by trained local female volunteers called Poshan Sakhis, who were supervised by Mentors. The duration of BCC implementation varied across the 8 treatment blocks. The third phase of NI BCC was a refresher subset of content from phases 1 and 2 to reinforce key messages. POSHAN Abhiyaan is the Government of India's National Nutrition Mission and was not part of the study design but was implemented in all study areas. BCC, behavior change communication; NI, nutrition-intensive; PRADAN, Professional Assistance for Development Action; SHG, self-help group.

PRADAN's standard activities include a dedicated cadre responsible for agriculture-focused discussions in SHG meetings (new tools/technologies, market linkages), leadership training, collective action, and awareness around government programs and broad gender issues. PRADAN's model is predicated on the belief that community change is driven primarily by members of the community themselves, who disseminate information and training received to those in their community who were not primary beneficiaries. Information dissemination between SHGs at higher levels is also expected, because multiple SHGs are federated into village organizations and multiple village organizations into a block level federation (**Figure 1**). In 2014, PRADAN partnered with the Public Health Resource Society (PHRS) to design and implement a “nutrition intensive” (NI) BCC intervention delivered to members of PRADAN's SHGs. Nutrition messages were delivered through an interactive storytelling process to women in 1 block per district at their monthly SHG meetings by a trained facilitator called a Poshan Sakhi, or “nutrition friend.” Poshan Sakhis were women from the community who were selected by their SHGs for their role; there were no requirements in terms of age, education, caste, or other characteristics for being a Poshan Sakhi. There was a preference for basic literacy among Poshan Sakhis, but this was not always possible. Each Poshan Sakhi had a Mentor, a paid PHRS staff member who provided support to the Poshan Sakhi. In each NI block, there was also 1 block program officer in charge of managing all NI activities and reporting back to PHRS. An A, B, C grading system was used such that “B” and “C” graded Poshan Sakhis were more actively supported by Mentors than “A” graded Poshan Sakhis. On average across study areas, 1 Mentor managed between 15 and 20 Poshan Sakhis, and 1 Poshan Sakhi delivered messages to between 2 and 4 SHGs in her hamlet or village, each SHG having 12–13 members on average.

The nutrition BCC messages were delivered through a storytelling process in 3 phases (Figure 1). Overall, with some variation across blocks, the interventions began in November 2016 and ended in July 2019. The process of content development began with a community needs assessment to understand the status and needs of the community members. The topics included in the resource material were decided based on the findings of the needs assessment exercise and 3 y of experience amongst similar communities (including in geographical areas common to this program) alongside consultations with program managers, field personnel, and experts in PLA on nutrition and health such as Ekjut. An initial pilot of the intervention also offered valuable insight into community preferences for receiving information and messages. Each phase contained a series of “micromodules,” which covered topics related to pregnancy, diet of women and children, agriculture, rights and entitlements, and so on (**Supplemental Table 1**). In addition to the interactive nutrition messaging at SHG meetings, each block team was encouraged to organize community events on health and nutrition such as BMI camps and cooking demonstrations.

Independent from the PHRS NI intervention evaluated by this study, the POSHAN Abhiyaan or National Nutrition Mission (NNM) was rolled out in 2017 and publicly launched by India's Prime Minister in early 2018. This flagship program to improve nutritional outcomes for children, pregnant women, and lactating mothers involves a mobile app and dashboard for health workers, convergence of nutrition actors across sectors, strengthened monitoring of nutrition and health service delivery, a behavior change campaign, and capacity building. The behavior change campaign, known as Jan Andolan, or “People's Movement,” uses multiple platforms (mass media, community events, and home visits) and covers multiple nutrition themes common to those covered in PHRS's NI intervention (nutrition across the life cycle, marriage timing, breastfeeding and complementary feeding, nutrition for pregnant women, anemia prevention, hygiene, etc.) (23). Poshan Maahs, or “Nutrition months,” which occurred in September 2018 and September 2019, were particularly intense periods of NNM implementation. By early 2019, the NNM had been rolled out in all 718 districts across India (24).

### Study design

The current study was conducted within a parent study, Women Improving Nutrition through Group-based Strategies (WINGS; 2015–2020), which aimed to measure the impact of PRADAN's standard agriculture-focused package of interventions and of the NI intervention on nutrition outcomes. The study was registered in 3ie's Registry for International Development Impact Evaluations (RIDIE-STUDY-ID-5d567e7e8b967; https://ridie.3ieimpact.org/). In the parent study, blocks in a district either received standard PRADAN interventions (STD group), or standard interventions plus the add-on nutrition BCC from PHRS (NI group), or no interventions (control non-PRADAN group). Interventions began in 2016. To address potential selection bias that might confound comparisons across groups, the 3 blocks (STD, NI, control) within each district were matched on demographic, economic, infrastructure, standard of living, and agriculture characteristics using data collected by PRADAN. Because the 2015 WINGS parent study panel survey of 2744 households only included ∼130 households per study arm with children aged 6–23.9 mo, we added cross-sectional surveys in 2017 and 2019 to better assess NI impacts on young child nutrition outcomes, a priority interest given the NI content. The current analysis uses data from the cross-sectional surveys in 2017 and 2019, noting that the cross-sectional “baseline” in 2017 was 12 mo after the start of the intervention; this feature is investigated and discussed below. Given the aim of understanding the additional benefit of the NI intervention beyond PRADAN's standard agriculture-focused model, the cross-sectional surveys were conducted in NI and STD blocks but not in control blocks where PRADAN was not operating. Within blocks, we randomly selected villages from a list of villages where PRADAN works and conducted a household census in these villages to identify eligible participants, repeating this process until the target sample size was achieved.

### Participants

All participants in the current study were women with children aged 6–23.9 mo living in NI and standard blocks. The only women with children aged 6–23.9 mo who were not invited to participate were those already enrolled in the parent panel study. SHG membership was not a criterion for participation because we sought to evaluate the impacts on all community members rather than only on program beneficiaries. This follows PRADAN's model of promoting sharing between direct beneficiaries and nonbeneficiaries.

### Outcomes

Nutrition outcomes included child dietary diversity and consumption of different food groups, child anthropometry [height-for-age, weight-for-age, and weight-for-height *z*-scores along with their corresponding binary outcomes of stunting, wasting, and underweight as well as midupper arm circumference (MUAC)], and maternal anthropometry (BMI, underweight, MUAC). Anthropometric data were collected using standard methods (25), and measurements were conducted by trained and standardized field staff. Weight was measured using digital weighing scales (OMRON) precise to 100 g, and height was measured using a standing stadiometer (SECA) for women or an infantometer (collapsible length/height boards with maximum length of 85 cm), which was precise to 1 mm, to measure children. MUAC was measured using a flexible measuring tape to the nearest 1 mm. Equipment was cleaned and calibrated at the beginning of each day of data collection. A gold standard supervisor revisited a random 5% of households to conduct repeated measurements for quality monitoring.

**FIGURE 2:**
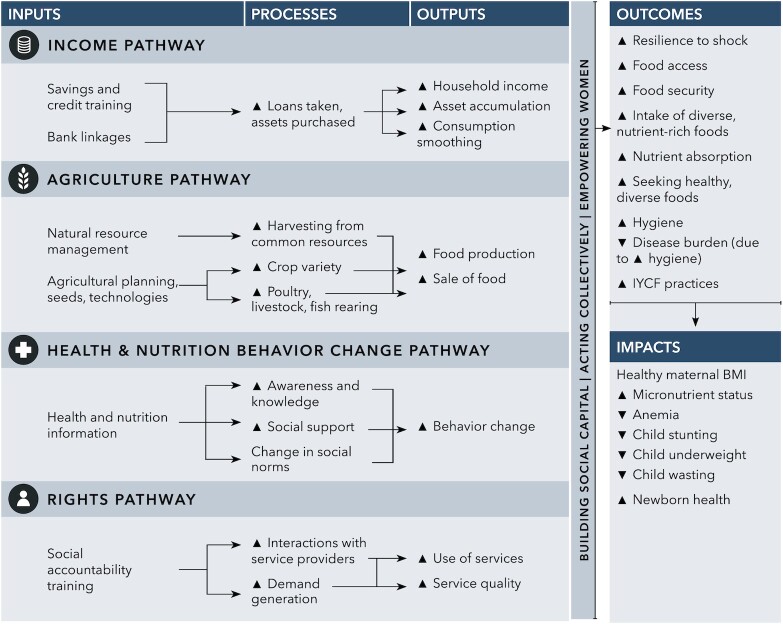
Conceptual pathways from women's groups to maternal and child nutrition outcomes. IYCF, infant and young child feeding. Originally published in Kumar et al., 2018 (19), published under a CC BY 4.0 license.

To guide our assessment of impacts of the NI intervention on intermediate factors along impact pathways, we utilized an existing conceptual framework for how interventions delivered through women's groups can achieve nutrition impacts in the South Asian context (**Figure 2**) (19). This framework proposed 4 parallel yet interlinked pathways—health- and nutrition-related behavior change, income generation, agriculture, and rights—as well as a cross-cutting pathway on women's empowerment. Indicators along each pathway and for all outcomes are defined in **Supplemental Table 2**. These included nutrition knowledge and health service utilization (health and nutrition BCC pathway), asset ownership and wealth (income pathway), food security and production diversity (agriculture pathway), awareness of government schemes for pregnant women (rights pathway), and women's decision-making ability related to her and her child's diet as well as progressive gender attitudes (empowerment pathway). The NI messaging included content related to all pathways, though primarily focused on the health and nutrition BCC pathway.

### Exposure measurement

Exposure to the intervention was measured using 4 approaches. At baseline, participants were asked if they had heard of “the story about [character name]” or the name of the main character in the stories delivered by Poshan Sakhis (approach 1) and if their SHG meeting ever included discussion on topics covered by the NI BCC intervention such as nutrition, breastfeeding, etc. (approach 2). At endline, we added questions to assess recall of topics covered in the stories such as early marriage and pregnancy registration. Hence, at endline, in addition to approaches 1 and 2, participants were asked if they had heard of “the story about [topic]” (approach 3). Finally, at endline, questions were added to determine potential exposure through community-level health and nutrition events such as BMI camps, food fairs, and village health meetings (approach 4).

### Sample size

Sample size was determined using data on infant and young child feeding (IYCF) practices and intraclass correlations from the 2015 parent panel survey. The clustersampsi Stata command (Stata version 16; StataCorp LLC) was used to determine the detectable effect size, assuming 8 clusters (blocks) per arm and 90 as a cluster size. The detectable effect sizes were satisfactory—between 0.23 SD and 0.38 SD for 4 different IYCF indicators. Thus, a sample size of 720 per treatment arm was determined and was increased to 800 per treatment arm for a total sample size of 1600 to account for potential data loss.

### Data collection

Data collection occurred in November 2017 to February 2018 (“baseline”) and December 2019 to March 2020 (“endline”). The 2019 round occurred in the same villages as the 2017 round, but on a repeated cross-sectional sample of households with children aged 6–23.9 mo. Data were collected in 1 of 3 languages (Hindi, Oriya, Bengali), depending on the study area, by enumerators from a professional survey firm, Oxford Policy Management, using Computer Assisted Personal Interviewing on tablets through the CSPro data entry platform. Survey questionnaires were pretested in control areas outside the evaluation areas. The translation of the final tools was shared with all study partners to perfect wording in the local languages. An extensive 20-d training including classroom and field components was led by Oxford Policy Management India's Survey Training and Quality Assurance specialist and attended by researchers from Oxford Policy Management and the International Food Policy Research Institute. Full details of field procedures and data quality checks are available in **Supplemental Table 3**.

### Statistical analysis

Prior to the collection of endline data, a preanalysis plan was submitted in the RIDIE system where the study was registered (see link above). To examine impacts of the NI intervention over and above impacts of the STD intervention, we followed the approach suggested by Blundell and Dias (26) wherein we built a pseudo panel and matched the NI households at endline to 3 separate groups: NI at baseline, STD at baseline, and STD at endline. Matching was done within a command in Stata called diff, which estimates a single propensity score model that includes all 4 groups in which the treatment indicator is defined as 1 for all treated endline observations and 0 for all treated baseline, comparison endline, and comparison baseline observations (27). The diff command conducts the kernel matching using a specified set of matching characteristics from both rounds (matching covariates are shown in **Supplemental Table 4**), producing 3 sets of kernel weights for estimating the program impacts, and then estimates the difference-in-difference (DID) model treatment effects. We also included a predetermined set of covariates in all models that could be related to the outcomes but not to treatment allocation: district-level geographic dummies, woman's age and education, household dependency ratio, and household head caste. Disadvantaged caste types included scheduled tribe, scheduled caste, and other backward class. These caste types are official classifications of the Government of India and were conceptualized with the idea of needing to provide certain economic and social benefits to groups of people who have been historically oppressed in order to protect their rights and promote equity. For further explanation on India's caste types, see Gopinath (28). When examining child-level outcomes, we included child age and gender as additional covariates. In simpler terms, the DID model used in this analysis follows an intent-to-treat approach and tests the hypothesis that outcomes in the NI group improved more than outcomes in the STD group from baseline to endline. We reran the same DID model on a subsample of women living in households with ≥1 adult female SHG member; given a higher likelihood of exposure, treatment effects in this subsample might be more easily observed. All models used to measure impacts employed bootstrapped SEs. All analyses were performed using Stata version 16. Statistical significance was considered using a cutoff of *P* < 0.05.

### Ethical approval

The study underwent ethical review and received ethical approval from the institutional review boards of the International Food Policy Research Institute (United States) and Sigma Research and Consulting (India) in compliance with the Helsinki Declaration as revised in 1983. All study participants provided their informed consent and authorized the future use of their anonymized data for research purposes. Data were anonymized immediately after data collection. The principal investigator of the trial (NK) was responsible for the security of identifiable data. There were no benefits to participation and no compensation was provided.

## Results

### Sample description

Unmatched mean sample characteristics by treatment group for the 2 different surveys (baseline and endline) are shown for the full sample in **Table 1**. We achieved our target sample size of 800 per group other than at baseline with 776 in the NI group. On average across group and round, women were 26 y old, had 5 y of education, and were married when they were 18–19 y old. In the baseline sample, 43% of women were SHG members. Fewer women were SHG members in the endline sample (36%); although they had belonged to SHGs for slightly longer, they reported attending fewer meetings in the 3 mo preceding the interview. Children were aged 15 mo on average and half were male. Most (>80%) women and children were from Hindu, lower-caste/tribe households with electricity. About half of the households had ≥1 female SHG member; these households made up the subsample for our secondary DID analysis. There were statistically significant differences between NI and STD groups for certain characteristics within each survey—for example, at endline, women in the NI group were married slightly earlier and had belonged to an SHG slightly longer—but these differences were accounted for in the matching analysis (**Supplemental Table 5**).

**TABLE 1:** Baseline and endline household, mother, and child characteristics by treatment arm^1^

	Baseline			Endline		
	NI	Standard		NI	Standard	
	*n = *776	*n = *833	*P* value	*n = *885	*n = *966	*P* value
Mother						
Age, y	25.63 ± 4.57	25.48 ± 4.50	0.52	25.56 ± 4.86	25.96 ± 4.95	0.08*
Completed education, y	5.04 ± 4.37	4.87 ± 4.30	0.45	5.71 ± 4.34	5.34 ± 4.40	0.07*
Age at marriage	18.28 ± 2.34	18.33 ± 2.45	0.67	18.51 ± 2.65	18.89 ± 2.89	0.00***
Current SHG member, %	43.69	42.98	0.78	38.52	34.16	0.05*
How long belonged to SHG, mo	29.29 ± 30.75	26.93 ± 30.60	0.31	34.65 ± 36.26	29.64 ± 24.38	0.04**
Self-reported “active” SHG member, %	81.42	77.93	0.25	84.88	80.55	0.14
>6 SHG meetings attended in last 3 mo, %	49.40	45.53	0.31	43.58	36.53	0.07*
Child						
Age, mo	15.20 ± 5.06	15.41 ± 4.87	0.43	15.70 ± 4.78	15.36 ± 4.79	0.13
Male, %	55.10	53.86	0.64	48.70	49.22	0.83
Household						
HH size, no. of members	5.50 ± 1.76	5.55 ± 1.82	0.52	5.40 ± 1.81	5.36 ± 1.80	0.66
Ratio of male to female HH members	1.13 ± 0.83	1.17 ± 0.83	0.39	1.07 ± 0.79	1.09 ± 0.80	0.61
Dependency ratio (<15 y + >55 y:16–55 y)	1.08 ± 0.67	0.99 ± 0.70	0.01**	1.04 ± 0.68	1.11 ± 0.74	0.04**
HH head religion: Hindu, %	87.37	87.15	0.90	88.13	88.41	0.85
HH head caste: scheduled caste,^2^ %	15.59	12.73	0.10*	8.62	4.76	0.00***
HH head caste: scheduled tribe,^2^ %	54.12	66.51	0.00***	58.45	72.98	0.00***
HH head caste: OBC,^2^ %	26.80	18.73	0.00***	28.11	20.39	0.00***
Owns house, %	98.71	98.68	0.96	98.77	98.76	0.98
Has electricity, %	83.51	81.99	0.42	94.06	93.48	0.60
Uses improved drinking water source, %	68.94	73.71	0.04**	67.41	72.57	0.01**
Main roof made of improved material, %	14.56	15.85	0.47	29.90	31.26	0.52
Main floor made of improved material, %	23.45	19.33	0.04**	29.79	21.53	0.00***
Main exterior wall made of improved material, %	35.44	32.53	0.22	46.25	42.13	0.07*
Any HH member has bank account, %	95.10	94.00	0.33	98.43	96.89	0.03**
Highest completed education for HH females, y	8.08 ± 12.10	7.61 ± 11.43	0.42	6.41 ± 4.08	6.05 ± 4.20	0.06*
≥1 female SHG member aged >15 y, %	53.22	53.54	0.90	50.17	47.41	0.24
Any crop/livestock/poultry loss in last 1 y, %	37.98	35.71	0.35	46.97	50.89	0.09*
New birth in last 1 y, %	31.06	30.29	0.74	41.03	42.99	0.39
Death in last 1 y, %	5.67	6.12	0.70	5.27	5.91	0.55

1 Group means (mean ± SD or percentage) are shown for the unmatched full sample. Level of significance for difference between treatment arms: *P < 0.10; **P < 0.05; ***P < 0.01. HH, household; NI, nutrition intensive; OBC, other backward class; SHG, self-help group.

2 Scheduled caste, scheduled tribe, and other backward class are official designations of the Government of India used to identify groups of individuals who have historically been disadvantaged economically and socially. For further description of these caste types, see Gopinath 2018 (28).

### Exposure to the intervention in the full sample

At baseline, 12 mo after the BCC intervention started, <10% of women in the NI group were able to openly recall stories involving different characters without prompting on the story topics; though low, exposure was higher in the NI group compared with the STD group (**Table 2**). When asked about topics discussed at SHG meetings, 20–50% of women in both arms reported that nutrition, child feeding, and agriculture had been discussed. At endline, the questionnaire was expanded to measure exposure to stories about key BCC topics rather than character names; however, only ∼10% of women in the NI group had heard stories about these topics, along with a small percentage (1–7%) in the STD group, suggesting an alternate source of BCC messaging. Further, very few women reported ever participating in health- and nutrition-related community events at endline. In terms of SHG meeting discussion topics, exposure was slightly higher in the endline sample compared with baseline for both groups, especially for breastfeeding, child feeding, water, sanitation, and hygiene, rights and entitlements, and treatment of women. However, the percentage of women who discussed these topics was similar in NI and STD groups.

**TABLE 2:** Baseline and endline intervention exposure by treatment arm^1^

	Baseline			Endline		
	NI	Standard		NI	Standard	
	*n *= 776	*n *= 833	*P* value	*n *= 885	*n *= 966	*P* value
Heard story about…, %						
Character: Soni	7.56	0.36	0.00***	8.90	1.66	0.00***
Character: Madhu	6.79	0.24	0.00***	8.12	1.66	0.00***
Character: Silvanti	2.77	0.12	0.00***	5.65	0.62	0.00***
Topic: Early marriage/pregnancy	—	—	—	10.02	3.11	0.00***
Topic: Pregnancy registration at AWC	—	—	—	16.48	6.63	0.00***
Topic: Growing food in home garden	—	—	—	7.68	1.97	0.00***
Topic: Women's anemia	—	—	—	7.01	1.66	0.00***
Topic: Delivery and small family	—	—	—	6.66	1.45	0.00***
Topic: Taking care of newborn	—	—	—	9.98	1.76	0.00***
Topic: Taking care of child older than 6 mo	—	—	—	9.92	1.35	0.00***
Topic: Voting and entitlements	—	—	—	11.09	4.26	0.00***
Ever participated in…, %						
BMI camp	—	—	—	7.27	4.60	0.01**
Hemoglobin camp	—	—	—	6.98	4.91	0.06*
Health/nutrition fair	—	—	—	2.75	0.52	0.00***
Nutritious food fair	—	—	—	1.38	0.42	0.03**
Village health and nutrition meeting	—	—	—	3.32	1.36	0.01***
Large health meeting	—	—	—	1.14	0.63	0.24
SHG meeting ever included discussion on…, %						
Nutrition	43.07	35.75	0.05**	43.90	45.45	0.69
Breastfeeding	25.07	20.39	0.14	34.88	31.52	0.35
Child feeding	23.01	19.55	0.27	36.63	35.76	0.82
WASH	46.02	40.50	0.14	49.71	52.73	0.43
Agriculture/livestock/poultry	57.52	49.72	0.04**	52.33	53.64	0.73
Rights and entitlements	23.30	21.51	0.57	35.17	35.45	0.94
Treatment of women	25.07	25.98	0.79	41.28	46.97	0.14
Education	42.18	37.99	0.26	34.01	38.48	0.23

1 All numbers are percentages. Group means (percentages) are shown for the unmatched full sample. Em dash denotes not measured. Level of significance for NI vs. standard comparison within timepoint: *P < 0.10; **P < 0.05; ***P < 0.01. SHG meeting discussion questions were only asked to the subsample of SHG members (∼40% of full sample). AWC, Anganwadi center; NI, nutrition intensive; SHG, self-help group; WASH, water, sanitation, and hygiene.

### Impacts of the NI intervention on diet and anthropometry

In the full sample, child feeding practices and child diet generally improved over time except for early breastfeeding initiation (**Table 3**). However, child diets were poor at both timepoints, with only 3–4 food groups consumed in the previous day, 14–23% achieving minimum dietary diversity, 16–26% consuming egg or flesh foods, and 80% consuming unhealthy foods such as biscuits, sweets, and instant noodles. Egg or flesh food consumption in children increased more in the NI compared with STD group (full sample DID 0.08, *P* = 0.01) and staple grain consumption increased less in the NI compared with STD group (full sample DID −0.05, *P* = 0.02) as expected. We consider the impact on egg and flesh food consumption meaningful given that the NI group improved from 16% to 26% whereas the STD group decreased from 20% to 18%. The impact on staple grain consumption was less meaningful because nearly all children consumed this food group. Child anthropometry was also poor with ∼55% of children being stunted, 45% underweight, and 20% wasted, but no impacts of the NI intervention were observed other than a small positive impact on weight-for-height *z*-score (subsample DID 0.31, *P* = 0.03) in the subsample of women from households with ≥1 SHG member.

**TABLE 3:** Intervention effects on diet and anthropometry in children aged 6–24 mo^1^

					DID with kernel matching			
	Baseline		Endline		Full sample		Subsample with ≥1 SHG member in HH	
	NI	Standard	NI	Standard	*n* = 3165		*n* = 1603	
	*n* = 776	*n* = 833	*n* = 885	*n* = 966	Estimate	*P* value	Estimate	*P* value
Feeding practices and diet								
Early BF initiation, %	54.44	49.62	42.26	45.35	−0.04 ± 0.04	0.26	−0.01 ± 0.06	0.91
Exclusive BF first 3 d, %	93.68	93.90	92.50	90.19	0.02 ± 0.02	0.33	0.04 ± 0.03	0.22
Continued BF (12–23 mo), %	94.44	95.65	94.98	97.63	−0.01 ± 0.02	0.76	0.02 ± 0.02	0.48
Food groups consumed, *n*	3.50 ± 1.10	3.43 ± 1.15	3.82 ± 1.09	3.74 ± 1.07	0.01 ± 0.08	0.90	−0.00 ± 0.13	0.97
Minimum diet diversity, %	15.21	13.87	22.84	19.79	0.01 ± 0.03	0.73	0.00 ± 0.05	0.96
Minimum meal frequency, %	75.00	73.95	74.14	75.21	−0.02 ± 0.03	0.48	−0.06 ± 0.05	0.24
Minimum acceptable diet, %	12.81	9.97	18.80	16.41	−0.02 ± 0.03	0.37	−0.03 ± 0.04	0.54
Egg or flesh food consumption, %	16.35	19.57	25.93	18.10	0.08 ± 0.03	0.01***	0.07 ± 0.04	0.11
Sweetened beverage consumption, %	22.56	22.75	22.74	21.21	0.01 ± 0.03	0.80	0.02 ± 0.05	0.62
Unhealthy food consumption, %	79.85	78.13	80.75	79.92	−0.02 ± 0.03	0.39	0.08 ± 0.03	0.03**
Zero fruits and vegetables, %	34.45	40.83	29.22	33.05	−0.00 ± 0.04	0.93	−0.00 ± 0.05	0.94
Individual food group consumed, %								
Breastmilk	95.14	96.01	96.04	97.60	−0.00 ± 0.02	0.97	0.01 ± 0.02	0.76
Grains, roots, or tubers	91.09	85.47	95.47	96.65	−0.05 ± 0.02	0.01**	−0.03 ± 0.03	0.26
Pulses (legumes and nuts)	69.64	70.49	71.35	73.85	−0.03 ± 0.04	0.46	−0.01 ± 0.05	0.77
Vitamin A rich–fruits and vegetables	64.96	58.10	69.42	65.48	0.00 ± 0.04	0.92	−0.01 ± 0.05	0.78
Other fruits	3.36	5.50	5.89	6.80	−0.00 ± 0.02	0.85	0.02 ± 0.03	0.62
Flesh foods (meat, fish, poultry)	8.91	12.84	15.18	11.09	0.06 ± 0.03	0.03**	0.06 ± 0.04	0.11
Egg	9.05	7.66	14.40	10.16	0.01 ± 0.02	0.76	−0.02 ± 0.03	0.65
Dairy products (milk, yogurt, cheese)	7.86	6.56	14.48	12.43	0.01 ± 0.02	0.54	−0.01 ± 0.03	0.77
Anthropometry								
Stunted, %	57.95	56.26	54.58	55.19	−0.06 ± 0.04	0.09*	−0.04 ± 0.05	0.43
HAZ	−2.20 ± 1.40	−2.24 ± 1.38	−2.12 ± 1.32	−2.11 ± 1.27	0.04 ± 0.10	0.67	−0.09 ± 0.14	0.53
Underweight, %	46.25	46.37	44.08	44.82	−0.01 ± 0.04	0.78	−0.07 ± 0.06	0.27
WAZ	−1.88 ± 1.17	−1.90 ± 1.12	−1.83 ± 1.13	−1.84 ± 1.13	−0.01 ± 0.09	0.89	0.14 ± 0.12	0.25
Wasted, %	20.07	20.79	18.42	17.85	0.02 ± 0.03	0.62	−0.05 ± 0.04	0.28
WHZ	−0.94 ± 1.40	−0.97 ± 1.25	−1.00 ± 1.19	−1.02 ± 1.16	−0.03 ± 0.10	0.81	0.31 ± 0.15	0.03**
MUAC, mm	119.66 ± 26.21	122.30 ± 52.26	138.02 ± 9.15	138.86 ± 28.88	2.22 ± 5.69	0.70	11.91 ± 15.80	0.45

1 Sample sizes are slightly smaller for anthropometry outcomes (*n* = 2862 to 3086 for full sample DID; *n* = 1455 to 1589 for subsample DID). Group means (mean ± SD or percentages) are shown for the unmatched full sample. Estimates from the DID include ± bootstrapped SEs, and DID model includes woman's age and education, household dependency ratio, household head caste, child age, child gender, and district of residence covariates. Level of significance of difference in DID estimate from zero: *P < 0.10; **P < 0.05; ***P < 0.01. BF, breastfeeding; DID, difference-in-difference model; HAZ, height-for-age *z*-score; HH, household; MUAC, midupper arm circumference; NI, nutrition intensive; SHG, self-help group; WAZ, weight-for-age *z*-score; WHZ, weight-for-height *z*-score.

Women consumed 3.6–4.0 food groups on average, and the percentage of women who achieved minimum dietary diversity was lower in the endline sample (∼20%) compared with the baseline sample (∼30%) (**Table 4**). Only one-third of women consumed any animal-source foods, but two-thirds consumed unhealthy foods. The NI intervention had a positive protective effect against a decline in consumption of nuts and seeds (full sample DID 0.04, *P* = 0.02) but a negative effect on the consumption of dark-green leafy vegetables (full sample DID −0.08, *P* = 0.04); neither of these impacts were statistically significant in the analysis of households with ≥1 SHG member. The positive impact on consumption of nuts and seeds is not very meaningful given that very few women consumed nuts and seeds (∼5% at baseline and ∼3% at endline). Underweight was common in women (40–50%) but we found no impacts of the NI intervention on women's BMI or underweight.

**TABLE 4:** Intervention effects on diet and anthropometry in women^1^

					DID with kernel matching			
	Baseline		Endline		Full sample		Subsample with ≥1 SHG member in HH	
	NI	Standard	NI	Standard	*n* = 3167		*n* = 1604	
	*n* = 776	*n* = 833	*n* = 885	*n* = 966	Estimate	*P* value	Estimate	*P* value
Diet								
Food groups consumed, *n*	3.98 ± 1.25	3.95 ± 1.34	3.71 ± 1.21	3.60 ± 1.20	−0.05 ± 0.10	0.65	−0.02 ± 0.16	0.89
Minimum dietary diversity, %	30.32	30.01	22.73	18.86	−0.00 ± 0.04	0.97	−0.01 ± 0.05	0.89
Individual food group consumed, %								
Staples	99.61	98.92	99.66	99.69	−0.00 ± 0.00	0.57	0.00 ± 0.00	0.67
Pulses	73.84	73.83	75.48	75.57	0.00 ± 0.03	0.95	−0.01 ± 0.05	0.87
Nuts and seeds	4.25	6.24	3.14	2.59	0.04 ± 0.02	0.02**	0.06 ± 0.03	0.08*
Milk and milk products	13.14	12.00	11.65	10.97	−0.04 ± 0.02	0.07*	−0.03 ± 0.04	0.40
Flesh	17.65	18.61	19.37	16.87	0.02 ± 0.03	0.44	0.04 ± 0.04	0.42
Eggs	6.70	8.04	9.85	7.25	0.02 ± 0.02	0.32	0.01 ± 0.03	0.82
Dark-green leafy vegetables	55.41	48.74	35.05	36.75	−0.08 ± 0.04	0.04**	−0.05 ± 0.05	0.31
Vitamin A–rich fruits and vegetables	31.70	29.41	21.72	15.73	−0.01 ± 0.03	0.83	−0.00 ± 0.05	0.95
Other vegetables	80.54	79.59	84.32	81.26	0.02 ± 0.03	0.59	0.01 ± 0.04	0.72
Other fruits	14.95	19.93	10.41	13.35	−0.01 ± 0.03	0.71	−0.04 ± 0.04	0.38
Animal-source food: flesh, eggs, milk	32.60	32.89	35.16	29.92	0.00 ± 0.04	0.97	0.02 ± 0.05	0.68
Unhealthy food: sweets, fried, sugar	67.01	63.27	70.10	64.80	−0.00 ± 0.03	0.92	0.01 ± 0.05	0.82
Anthropometry								
BMI, kg/m^2^	19.44 ± 2.62	19.74 ± 2.97	18.89 ± 2.33	18.93 ± 2.21	0.26 ± 0.22	0.23	0.29 ± 0.31	0.35
Underweight (BMI <18.5), %	42.46	38.88	47.57	46.04	−0.03 ± 0.04	0.49	−0.04 ± 0.05	0.40
MUAC, mm	218.67 ± 96.11	216.58 ± 36.01	231.23 ± 43.72	231.92 ± 78.47	−7.72 ± 4.81	0.11	−6.67 ± 8.43	0.43

1 Sample sizes are slightly smaller for anthropometry outcomes (*n* = 2942 to 2946 for full sample DID; *n* = 1507 to 1511 for subsample DID). Group means (mean ± SD or percentages) are shown for the unmatched full sample. Estimates from the DID include ± bootstrapped SEs, and DID model includes woman's age and education, household dependency ratio, household head caste, and district of residence covariates. Level of significance of difference in DID estimate from zero: *P < 0.10; **P < 0.05; ***P < 0.01. DID, difference-in-difference model; HH, household; MUAC, midupper arm circumference; NI, nutrition intensive; SHG, self-help group.

### Impacts of the NI intervention on impact pathway indicators

Women's knowledge of nutrition information delivered as part of the NI intervention ranged from 50 to 90 on a 100-point scale for different knowledge domains (**Table 5**). Knowledge of maternal health/nutrition and dietary diversity was higher than knowledge of child health/nutrition. However, knowledge unexpectedly improved more in the STD group compared with the NI group (full sample DID for knowledge of maternal health/nutrition −4.83, *P* = 0.02). In terms of maternal health service use, we found small positive impacts of the NI intervention on receipt of take-home rations during the breastfeeding period (full sample DID 0.07, *P* = 0.01) and, for the subsample, on clinical checks during antenatal care (subsample DID 0.4, *P* = 0.03) and receipt of take-home rations during pregnancy (subsample DID 0.08, *P* = 0.03).

**TABLE 5:** Intervention effects on pathway indicators: income, livelihoods, nutrition behavior, rights, empowerment^1^

					DID with kernel matching			
	Baseline		Endline		Full sample		Subsample with ≥1 SHG member in HH	
	NI	Standard	NI	Standard	*n* = 3167		*n* = 1604	
	*n* = 776	*n* = 833	*n* = 885	*n* = 966	Estimate	*P* value	Estimate	*P* value
Nutrition behavior pathway								
Knowledge—child feeding (0–100)	60.55 ± 17.98	59.05 ± 17.89	60.08 ± 16.10	57.63 ± 16.73	0.35 ± 1.28	0.79	−3.14 ± 1.68	0.06*
Knowledge—child health, hygiene (0–100)	53.99 ± 32.33	53.90 ± 31.50	69.73 ± 30.74	64.32 ± 32.88	4.12 ± 2.31	0.07*	−0.85 ± 3.23	0.79
Knowledge—maternal health/nutrition (0–100)	59.12 ± 29.43	55.61 ± 30.04	86.06 ± 21.48	84.86 ± 22.77	−4.83 ± 2.07	0.02**	−9.41 ± 2.78	0.00***
Knowledge—dietary diversity (0–100)	71.93 ± 28.90	69.68 ± 30.38	83.09 ± 24.63	80.81 ± 26.96	−2.12 ± 2.13	0.32	−7.85 ± 2.68	0.00***
Knowledge—overall (0–100)	62.05 ± 16.88	60.19 ± 17.57	71.89 ± 14.98	69.29 ± 16.66	−0.69 ± 1.22	0.57	−5.13 ± 1.59	0.00***
Registered pregnancy	96.13	94.11	99.10	98.45	−0.02 ± 0.01	0.18	−0.00 ± 0.02	0.84
Any ANC check-up	98.71	97.71	99.44	98.76	−0.01 ± 0.01	0.46	0.00 ± 0.01	0.77
≥4 ANC during pregnancy	62.99	59.03	73.49	68.33	−0.02 ± 0.04	0.49	−0.02 ± 0.05	0.68
ANC in 1st trimester	78.47	78.01	85.96	83.98	0.03 ± 0.03	0.34	0.05 ± 0.04	0.21
ANC quality—total score (0–1)	0.76 ± 0.21	0.74 ± 0.22	0.88 ± 0.15	0.83 ± 0.21	0.02 ± 0.01	0.14	0.02 ± 0.02	0.40
ANC quality—clinical score (0–1)	0.91 ± 0.22	0.89 ± 0.24	0.96 ± 0.12	0.92 ± 0.21	0.02 ± 0.01	0.22	0.04 ± 0.02	0.03**
ANC quality—advice score (0–1)	0.66 ± 0.26	0.63 ± 0.26	0.82 ± 0.22	0.76 ± 0.26	0.02 ± 0.02	0.38	0.00 ± 0.03	0.89
Institutional delivery	69.97	71.19	77.83	75.26	0.03 ± 0.03	0.29	0.04 ± 0.04	0.31
THR receipt—pregnancy period	90.46	90.25	76.48	78.45	0.04 ± 0.02	0.11	0.08 ± 0.04	0.03**
THR receipt—BF period	90.68	90.60	70.03	69.74	0.07 ± 0.03	0.01**	0.08 ± 0.04	0.04**
THR receipt—CF feeding period	85.38	83.92	74.92	76.50	0.04 ± 0.03	0.16	0.04 ± 0.04	0.28
Income pathway								
Type of assets owned by HH, *n*	6.51 ± 3.15	6.69 ± 3.10	7.43 ± 3.19	7.24 ± 3.11	0.02 ± 0.22	0.92	0.25 ± 0.39	0.51
Wealth index	−0.04 ± 2.06	0.05 ± 2.11	0.09 ± 2.14	−0.09 ± 2.04	0.08 ± 0.16	0.62	0.24 ± 0.31	0.44
Agriculture/livelihoods pathway								
HFIAS score (0–27)	2.53 ± 3.39	2.45 ± 3.42	3.21 ± 3.96	3.19 ± 3.99	0.08 ± 0.28	0.77	0.15 ± 0.36	0.68
Food secure	48.97	52.22	46.02	47.31	−0.00 ± 0.04	1.00	0.01 ± 0.05	0.84
Production diversity index (0–10)	1.35 ± 1.21	1.23 ± 1.24	1.72 ± 1.59	1.73 ± 1.53	−0.05 ± 0.08	0.52	−0.06 ± 0.12	0.65
Rights pa*t*hway								
Awareness—JSY, %	93.81	92.20	98.10	96.07	−0.01 ± 0.02	0.38	−0.02 ± 0.02	0.42
Awareness—JSSK, %	66.88	61.10	82.19	80.23	−0.04 ± 0.03	0.20	−0.03 ± 0.04	0.53
Enrolment—JSY, %	75.49	70.72	84.23	81.75	−0.02 ± 0.03	0.45	−0.04 ± 0.04	0.37
Enrolment—JSSK, %	47.66	42.01	62.90	57.83	−0.03 ± 0.03	0.31	−0.05 ± 0.05	0.29
Empowerment pathway								
Decision-making—women's diet, %	88.40	88.00	88.91	91.30	−0.01 ± 0.02	0.54	−0.01 ± 0.03	0.78
Decision-making—diet during pregnancy, %	76.16	75.96	76.26	79.50	0.00 ± 0.03	0.97	0.01 ± 0.05	0.86
Decision-making—child's diet, %	81.94	80.77	84.44	86.01	0.05 ± 0.03	0.07*	0.07 ± 0.04	0.06*
Decision-making—BF and CF, %	88.02	85.71	84.99	84.46	0.04 ± 0.03	0.15	0.06 ± 0.04	0.16
Progressive gender attitude score (0–1)	0.73 ± 0.24	0.75 ± 0.22	0.75 ± 0.20	0.76 ± 0.21	−0.01 ± 0.02	0.69	0.03 ± 0.02	0.21

1 Group means (mean ± SD or percentages) are shown for the unmatched full sample. Estimates from the DID include ± bootstrapped SEs, and DID model includes woman's age and education, household dependency ratio, household head caste, and district of residence covariate. Level of significance of difference in DID estimate from zero: *P < 0.10; **P < 0.05; *** *P* < 0.01. ANC, antenatal care; BF, breastfeeding; CF, complementary feeding; DID, difference-in-difference model; HFIAS, household food insecurity access scale; HH, household; JSY, Janani Suraksha Yojana; JSSK, Janani Shishu Suraksha Karyakaram; NI, nutrition intensive; SHG, self-help group; THR, take-home ration.

In terms of the other impact pathways, we first note that women were from poor households; half were food secure and only 1–2 crop categories were cultivated on average, but we found no evidence that the NI intervention benefited indicators along income or agriculture pathways. Most women were aware of Janani Suraksha Yojana and Janani Shishu Suraksha Karyakaram, two maternity benefit schemes, and awareness and enrolment in these schemes increased over time for both NI and STD groups, with no DID impacts. Approximately 80–90% of women were able to make their own decisions related to their diet and their child's diet, though decision-making ability did not increase over time and we found no evidence that the NI intervention benefited these outcomes. Similarly, these data do not support a benefit of the NI intervention on women's progressive gender attitudes.

## Discussion

We found limited impacts of a 3-y nutrition BCC intervention through agriculture-focused SHGs on nutrition outcomes and on intermediate pathway indicators expected to lead to those outcomes. Despite these limited impacts, much can be learned from the studied effort to improve women's nutrition through SHGs.

### Contrast with other group-based nutrition interventions

The evidence on the impact of group-based interventions to improve nutrition outcomes comes from a diverse set of groups: women's savings and credit-based SHGs, as in this study, but also other types of women's groups aimed at community mobilization, including groups open to the community and groups closed to specific sections of women (7). With some exceptions (11, 12, 29), evaluations of group-based interventions have focused on outcomes among group members, making their results somewhat hard to compare with the population-based estimates we present here. However, the overall limited impact we report is in line with the broader literature. Nutrition information or BCC interventions have shown positive impacts on self-reported dietary diversity, behavior linked to prevention of child illness, and select IYCF practices (11, 12, 17, 30), but limited impacts on anthropometry, even when combined with in-kind transfers (31). Even when only group members were studied, interventions layered onto existing at-scale SHG programs achieved low implementation intensity, either because messages were tailored to women younger than the average group member (17, 22, 32), or because other interventions delivered simultaneously through the same platform diluted impacts (33). Nutrition interventions leveraging community mobilization groups that relied on PLA or similar approaches achieved higher intensity. One study (12) involving members and nonmembers reported an impact on anthropometry–a sizeable reduction in child wasting—but combined PLA approaches with home visits and crèches that provided nutritious food for young children.

### Understanding exposure levels

We included specific and general measures of exposure and found that exposure to specific stories was low—∼10% at endline in the NI group and 3% in the STD group in the full sample (in SHG members, these percentages were 10% and 5% for NI and STD groups, respectively)—but 30–50% of SHG members in both NI and STD groups reported ever discussing general topics related to nutrition, agriculture, education, and gender in their SHGs. Four points can be made related to low exposure. First, it should be noted that the evaluation was designed to assess real-world impacts of a community-based intervention where women in intervention communities were expected to attend SHG meetings and share what they learned with their family and peers, thus we sampled women regardless of their membership status. Low exposure in our sample likely reflects implementation barriers and uptake barriers as well as limited diffusion from SHG members to nonmembers. Second, the relatively high exposure to general discussion topics among SHG members in both NI and STD groups suggests that PRADAN SHGs already cover thematic areas that were part of the NI intervention, which could have limited our ability to detect differences in outcomes between groups. Third, given limited SHG membership and limited exposure, the sample size was insufficient to run impact models only on the exposed group, hence our strategy of focusing on households with ≥1 SHG member for secondary impact analysis. Fourth, the PHRS model of improving community-level nutrition outcomes might require a longer timeframe to evaluate. This evaluation was during a learning phase, and full program reach might require several more years of implementation.

### Interpretation of findings in the context of India's NNM

The thematic and chronological overlap of PHRS's NI BCC intervention and the NNM might explain some of our findings. There was a 10–15 percentage point increase from baseline to endline in the percentage of women in the STD group who reported their SHGs ever discussed nutrition, breastfeeding, and child feeding. Knowledge of maternal health and nutrition, child health and hygiene, and dietary diversity also increased by 10–30 percentage points in the STD group. These secular trends are suggestive of an information campaign such as NNM reaching women in nonintervention blocks during the 2-y period between surveys. NNM might have been particularly well taken up in areas with existing health-focused civil society organizations operating on the ground, such as in the studied PRADAN blocks. We observed a few cases where Poshan Sakhis would use NNM events as opportunities to deliver NI messages to members of the larger community. This might also explain some exposure in the STD group. The scale of NNM far outweighs the studied NI intervention, with NNM having a workforce of 16 million including frontline workers, youth, and celebrity influencers. A large-scale media campaign utilizing social media, print, television, radio, and public spaces (paintings, performances) as platforms for message delivery is also financed under NNM. We do note from our exposure data that few women in both study arms reported participating in community events related to health and nutrition, but NNM could have reached women through other channels such as frontline workers or mass media. In terms of content, NNM covers 12 health- and nutrition-related themes, but 1 notable content gap is around consumption of animal-sourced foods in children. PHRS's NI intervention focused on age-appropriate animal-sourced food consumption in young children, especially during the revision module prior to endline, and results from our DID models identified a significant NI impact on child animal-source food consumption in the full sample. We also note the generally poor diets of children in this sample, with one-third of children consuming zero fruits and vegetables and 80% consuming unhealthy foods. These findings underscore the urgent need to refresh India's dietary data to better inform NNM planning, particularly in the context of the increasing double burden of undernutrition and overnutrition in India (34) and no national dietary surveys since 2011–2012 (35).

### Implementation barriers that could explain limited impacts

Our parallel qualitative research during the initial year of the BCC rollout in 3 of the 5 states (Jharkhand, Odisha, Madhya Pradesh) included in the evaluation supports our observations of limited impacts, identifying unrelatable or unactionable content, competing time demands of volunteers, low attendance at meetings, and limited capacity to supervise volunteers as barriers to change (36, 37). In particular, this work identified variable attendance at SHG meetings, particularly among women with small children, as a primary barrier for greater content exposure. Furthermore, the rapid increase in SHGs under NRLM meant that many members experienced neither sufficient material nor social benefits (e.g., in low-interest loans, livelihood improvements, mentorship) from SHG participation to justify staying attentive for information-only health interventions that occurred after core saving-lending activities. There were additional barriers in the dissemination of content as volunteers had variable levels of “soft-skills” training and were not always able to deliver material in a participatory, engaging manner as intended by program designers. Volunteers who were less confident in facilitating participatory discussion resorted to didactic information transfer, which SHG members were less attentive to. Though program curriculum was wide ranging and responsive to community-identified problems, the breadth of topics covered in a short 2-y timeframe also could have hindered a deeper engagement by SHG members, many of whom are occupied in multiple forms of household, agricultural, and wage labor.

### Strengths and limitations

Our study included a breadth of measures along impact pathways in addition to maternal and child diet and anthropometric outcomes. Study locations included blocks across 5 states in central and eastern India where the SHG movement has been active for many years and where there is much scope for improvement in nutrition. Building on an existing platform by adding a nutrition-specific information-only intervention is relevant to the current policy discourse around how SHGs can be used to address undernutrition in rural communities. We are fairly confident that low exposure reflects limited reach of the intervention rather than poor measurement. The design of specific measures of exposure at endline was a collaborative effort between evaluators and program implementers resulting from an acknowledgment of potentially poor measurement at baseline; it was felt that the character names might not be easy to recall, so we developed additional questions with wording on story content or topics. In addition, importantly, the model being tested was not a tightly controlled RCT but rather an assessment of a real-world program implemented by female volunteers across multiple geographies. We studied community-level impacts rather than impacts only on beneficiaries. Therefore, the study is pertinent to current efforts to understand how a large-scale program such as NRLM can be harnessed to deliver better nutrition to individuals living in high-poverty communities, not necessarily just SHG members, without high cost.

We acknowledge the following limitations of our evaluation. First, our study was not an RCT, thus we used a quasi-experimental design to address the challenges of selection bias. We used detailed measurement and robust statistical analyses to address a range of biases; we believe this provides a real-world example of a high-quality evaluation of a complex program that operated across several districts and states. Second, we recognize that the intervention began in November 2016, ∼12 mo before the first cross-sectional survey and thus it is possible that the NI group could have already experienced benefits by the time of the November 2017 “baseline” cross-sectional survey. We feel this is very unlikely given low levels of exposure at baseline. We also found low exposure to the intervention and almost no group differences in nutrition outcomes by 2017 in the survey data from the separate household panel survey that was part of the parent evaluation (**Supplemental Table 6**). The first year of the BCC intervention was still in a learning phase, with implementers refining the delivery and content.

### Conclusion

The SHG movement in India has undeniably benefited millions of women from poor households in multiple ways including connecting them to services and providing access to microcredits. Unlocking the potential of women's groups to deliver nutrition benefits, however, requires well-resourced and intensive, participatory interventions delivered by trained cadres with high capacity to the right individuals and their families before and during nutritionally vulnerable periods. Information-only interventions in disadvantaged settings continue to face steep systematic barriers to effectiveness such as resource scarcity, poor infrastructure, and limited opportunities for income generation. We agree with Raghunathan and Desai (8) who highlight 2 key areas of focus: *1*) effective intervention approaches, including both better pedagogical methods as well as improved tailoring of program content to target audiences, and *2*) a simultaneous emphasis on improving the ability of individuals to act on the newly acquired information, such as improving the supply of services and schemes that facilitate the adoption of recommended behavior, or improving individual and household resources through cash or in-kind transfers. Amidst the backdrop of a centrally financed national nutrition campaign, the currently studied information-only nutrition intervention delivered by local female volunteers had limited success in achieving desired impacts on maternal and child nutrition outcomes at the population level.

## Supplementary Material

nzac079_Supplemental_File

## Data Availability

Data described in the manuscript, code book, and analytic code will be made publicly and freely available without restriction at https://dataverse.harvard.edu/dataverse/IFPRI.

## Notes

This work was supported by the Bill & Melinda Gates Foundation (grant number OPP1132181). The funder had no role in the design, implementation, analysis, or interpretation of the data.

Author disclosures: VP and AH were employed by the Public Health Resource Society (PHRS). AC and MK were employed by Professional Assistance for Development Action (PRADAN). PHRS designed the nutrition education content, and PHRS and PRADAN co-implemented the intervention but had no influence on or involvement in the statistical analysis. The contributions of VP, AH, AC, and MK to this manuscript included written inputs to sections on intervention design, critical discussions regarding interpretation of results, and written review of manuscript drafts. Final decisions about results to include, interpretations, and conclusions rested with authors from the evaluation team (SS, SG, PM, KR, GT, AQ, NK). All other authors report no conflicts of interest.

Supplemental Tables 1–6 are available from the “Supplementary data” link in the online posting of the article and from the same link in the online table of contents at https://academic.oup.com/cdn/.

Abbreviations used: BCC, behavior change communication; DID, difference-in-difference; IYCF, infant and young child feeding; JEEViKA-MC, JEEViKA Multisectoral Convergence; MUAC, midupper arm circumference; NI, nutrition intensive; NNM, National Nutrition Mission; NRLM, National Rural Livelihood Mission; PHRS, Public Health Resource Society; PLA, participatory learning and action; PRADAN, Professional Assistance for Development Action; RCT, randomized controlled trial; SHG, self-help group; STD, standard arm; WINGS, Women Improving Nutrition through Group-based Strategies.

## References

[1] Victora CG , Christian P, Vidaletti LP, Gatica-Domínguez G, Menon P, Black RE. Revisiting maternal and child undernutrition in low-income and middle-income countries: variable progress towards an unfinished agenda. Lancet. 2021;397:1388–99. 33691094 10.1016/S0140-6736(21)00394-9 PMC7613170

[2] Chatterjee P . India's child malnutrition story worsens. Lancet Child Adolesc Health. 2021;5:319–20.

[3] Headey D , Heidkamp R, Osendarp S, Ruel M, Scott N, Black R et al. Impacts of COVID-19 on childhood malnutrition and nutrition-related mortality. Lancet. 2020;396(10250):519–21. 32730743 10.1016/S0140-6736(20)31647-0 PMC7384798

[4] Keats EC , Das JK, Salam RA, Lassi ZS, Imdad A, Black RE et al. Effective interventions to address maternal and child malnutrition: an update of the evidence. Lancet Child Adolesc Health. 2021;4642:1–18. 10.1016/S2352-4642(20)30274-1 33691083

[5] NRLM . National Rural Livelihood Mission – SHG analytical report. [Internet]. 2021; [cited 26 September, 2021]. Available from: https://nrlm.gov.in/shgOuterReports.do?methodName=showShgreport

[6] Reshmi SR , Dinachandra K, Bhanot A, Unisa S, Menon GT, Agrawal N et al. Context for layering women's nutrition interventions on a large scale poverty alleviation program: evidence from three eastern Indian states. PLoS One. 2019;14:e0210836. 30668595 10.1371/journal.pone.0210836 PMC6342298

[7] Desai S , Misra M, Das A, Singh RJ, Sehgal M, Gram L et al. Community interventions with women's groups to improve women's and children's health in India: a mixed-methods systematic review of effects, enablers and barriers. BMJ Glob Health. 2020;5:e003304. 10.1136/bmjgh-2020-003304 PMC7745316 33328199

[8] Raghunathan K , Desai S. Working with women's groups to improve nutrition in India. Econ Polit Wkly. 2021;56:17–21.

[9] Roy SS , Mahapatra R, Rath S, Bajpai A, Singh V, Rath S et al. Improved neonatal survival after participatory learning and action with women’s groups: a prospective study in rural eastern India. Bull World Health Organ. 2013;91(6):426–33B. 24052679 10.2471/BLT.12.105171 PMC3777144

[10] Tripathy P , Nair N, Barnett S, Mahapatra R, Borghi J, Rath S et al. Effect of a participatory intervention with women's groups on birth outcomes and maternal depression in Jharkhand and Orissa, India: a cluster-randomised controlled trial. Lancet. 2010;375(9721):1182–92. 20207411 10.1016/S0140-6736(09)62042-0

[11] Nair N , Tripathy P, Sachdev HS, Pradhan H, Bhattacharyya S, Gope R et al. Effect of participatory women's groups and counselling through home visits on children's linear growth in rural eastern India (CARING trial): a cluster-randomised controlled trial. Lancet Glob Health. 2017;5(10):e1004–16. 28911749 10.1016/S2214-109X(17)30339-X PMC5640793

[12] Gope RK , Tripathy P, Prasad V, Pradhan H, Sinha RK, Panda R et al. Effects of participatory learning and action with women's groups, counselling through home visits and crèches on undernutrition among children under three years in eastern India: a quasi-experimental study. BMC Public Health. 2019;19(1):962. 31319828 10.1186/s12889-019-7274-3 PMC6637592

[13] Kadiyala S , Harris-Fry H, Pradhan R, Mohanty S, Padhan S, Rath S et al. Effect of nutrition-sensitive agriculture interventions with participatory videos and women's group meetings on maternal and child nutritional outcomes in rural Odisha, India (UPAVAN trial): a four-arm, observer-blind, cluster-randomised controlled trial. Lancet Planet Health. 2021;5(5):e263–76. 33811818 10.1016/S2542-5196(21)00001-2 PMC8099729

[14] Prost A . Women's groups practising participatory learning and action to improve maternal and newborn health in low-resource settings: a systematic review and meta-analysis. Lancet. 2013;381(9879):1736–46. 23683640 10.1016/S0140-6736(13)60685-6 PMC3797417

[15] World Health Organization . WHO recommendation on community mobilization through facilitated participatory learning and action cycles with women's groups for maternal and newborn health. [Internet]. Geneva, Swizerland: WHO; 2014; [cited 26 September, 2021]. Available from: https://apps.who.int/iris/handle/10665/127939. 25165805

[16] Raghunathan K , Kumar N, Gupta S, Chauhan T, Kathuria AK, Menon P. Learning together: experimental evidence on the impact of group-based nutrition interventions in rural Bihar. IFPRI Discussion Paper 1936. Washington (DC): International Food Policy Research Institute; 2020.

[17] Hazra A , Atmavilas Y, Hay K, Saggurti N, Verma RK, Ahmad J et al. Effects of health behaviour change intervention through women's self-help groups on maternal and newborn health practices and related inequalities in rural India: a quasi-experimental study. EClinicalMedicine. 2020;18:100198. 31993574 10.1016/j.eclinm.2019.10.011 PMC6978187

[18] Pérez-Escamilla R , Odle J. Implementation science in the field of nutrition: why is it so relevant?. Curr Dev Nutr. 2018;3(3):nzy086. 30864561 10.1093/cdn/nzy086 PMC6400592

[19] Kumar N , Scott S, Menon P, Kannan S, Cunningham K, Tyagi P et al. Pathways from women's group-based programs to nutrition change in South Asia: a conceptual framework and literature review. Glob Food Security. 2018;17:172–85. 10.1016/j.gfs.2017.11.002 PMC6004534 29930896

[20] Ruel MT , Quisumbing AR, Balagamwala M. Nutrition-sensitive agriculture: what have we learned so far?. Glob Food Security. 2018;17:128–53.

[21] Gillespie S , Poole N, van den Bold M, Bhavani RV, Dangour AD, Shetty P. Leveraging agriculture for nutrition in South Asia: what do we know, and what have we learned?. Food Policy. 2019;82:3–12.

[22] Kochar A , Barooah B, Shah R. Findings from evaluation of National Rural Livelihoods Program, evidence-informed policymaking for rural transformation, New Delhi; 2020. Available from: https://www.3ieimpact.org/events/conferences/evidence-informed-policymaking-rural-transformation-exploration-role-womens

[23] World Bank . India's POSHAN Abhiyaan: behaviour change communication and community mobilisation for improved nutrition outcomes. [Internet]. 2019; [cited 26 September, 2021]. Available from: http://pubdocs.worldbank.org/en/146341582314669882/Note-4-Behavior-Change-Communication.pdf

[24] Ministry of Women and Child Development Government of India . National Nutrition Mission press release. [Internet]. 2019; [cited 26 September, 2021]. Available from: https://pib.gov.in/newsite/PrintRelease.aspx?relid=192306

[25] Cogill B . Anthropometric indicators measurement guide. Washington (DC): Food and Nutrition Technical Assistance Project, Academy for Educational Development; 2003.

[26] Blundell R , Dias MC. Alternative approaches to evaluation in empirical microeconomics. J Hum Resour. 2009;44:565–640.

[27] Villa JM . diff: symplifying the estimation of difference-in-differences treatment effects. Stata J. 2016;16:52–71.

[28] Gopinath V . Who are scheduled castes, scheduled tribes, other backward classes, and economically backward classes in India?. [Internet]. 2018; [cited March 23, 2022]. Available from: https://www.thequint.com/explainers/scheduled-caste-scheduled-tribe-obc-ebc-sc-st-prevention-of-atrocities-act-explainer#read-more

[29] Ojha S , Szatkowski L, Sinha R, Yaron G, Fogarty A, Allen SJ et al. Rojiroti microfinance and child nutrition: a cluster randomised trial. Arch Dis Child. 2020;105(3):229–35. 31601571 10.1136/archdischild-2018-316471 PMC7041497

[30] Saha S , Kermode M, Annear PL. Effect of combining a health program with a microfinance-based self-help group on health behaviors and outcomes. Public Health. 2015;129(11):1510–18. 26304181 10.1016/j.puhe.2015.07.010 PMC4652626

[31] Deininger K , Liu Y. Economic and social impacts of an innovative self-help group model in India. World Dev. 2013;43:149–63.

[32] Saggurti N , Atmavilas Y, Porwal A, Schooley J, Das R, Kande N et al. Effect of health intervention integration within women's self-help groups on collectivization and healthy practices around reproductive, maternal, neonatal and child health in rural India. PLoS One. 2018;13(8):e0202562. 30138397 10.1371/journal.pone.0202562 PMC6107172

[33] Avula R , Raghunathan K, Chauhan T. The Jeevika Multisectoral convergence pilot in Bihar: a process evaluation report. [Internet]. World Bank; 2019; [cited 26 September, 2021]. Available from: https://documents.worldbank.org/en/publication/documents-reports/documentdetail/396351572413728658/the-jeevika-multisectoral-convergence-pilot-in-bihar-a-process-evaluation-report

[34] Nguyen PH , Scott S, Headey D, Singh N, Tran LM, Menon P et al. The double burden of malnutrition in India: trends and inequalities (2006–2016). PLoS One. 2021;16(2):e0247856. 33630964 10.1371/journal.pone.0247856 PMC7906302

[35] National Nutrition Monitoring Bureau . Diet and nutritional status of rural population: report of third repeat survey. [Internet]. Hyderabad; 2012; [cited 26 September, 2021]. Available from: https://www.nin.res.in/downloads/NNMB_Third_Repeat_Rural_Survey%20%20%20Technicl_Report_26%20(1).pdf

[36] Nichols C . Spaces for women: rethinking behavior change communication in the context of women's groups and nutrition-sensitive agriculture. Soc Sci Med. 2021;285:114282. 34375897 10.1016/j.socscimed.2021.114282 PMC8434409

[37] Nichols C . Self-help groups as platforms for development: the role of social capital. World Dev. 2021;146:105575. 34602707 10.1016/j.worlddev.2021.105575 PMC8350316

