# Supplementary material for: A Quasi-Experimental Evaluation of a Nutrition Behavior Change Intervention Delivered Through Women's Self-Help Groups in Rural India: Impacts on Maternal and Young Child Diets, Anthropometry, and Intermediate Outcomes
Source: Curr Dev Nutr. 2022 Apr 11;6(6):nzac079. doi: 10.1093/cdn/nzac079 (PMC9177383; doi:10.1093/cdn/nzac079)
Supplement: nzac079_Supplemental_File [file nzac079_supplemental_file.docx]

| **Supplemental Table 1. Overview of topics covered in nutrition curriculum delivered through women’s SHGs** | |
| --- | --- |
| *Module* | *Topics covered* |
| Perspective building module 1 |  |
| MM 1 | Early marriage, rest during pregnancy, antenatal care, safe delivery, entitlements for pregnant women (cash or food assistance) |
| MM 2 | Colostrum feeding, breastfeeding, child illness management, complementary feeding, sanitation and hygiene |
| MM 3 | Women not eating last, diet diversity (eating tri-color meal), consuming animal-source foods, cooking in iron pot |
| MM 4 | Year-round kitchen gardens, types of foods to grow in different seasons |
| Perspective building module 2 |  |
| MM 5 | Women’s health and illness, anemia, malaria |
| MM 6 | Birth preparedness, family planning, maternity entitlements |
| MM 7 | Neonatal care, colostrum feeding, exclusive breastfeeding |
| MM 8 | Complementary feeding (timing, frequency, types of foods to give), childhood pneumonia |
| MM 9 | Rights and entitlements – anganwadi center, public distribution system, mid-day meal, maternity entitlements |
| Revision modules |  |
| RM 1 | Women not eating last, nutritious foods, vitamins and minerals, diet diversity (tri-color meal) |
| RM 2 | Kitchen gardens, foraging, PDS, foods to grow in different seasons, consuming less processed foods, alcohol, and tobacco |
| RM 3 | Appropriate BF/CF practices (colostrum, early initiation, positioning baby, frequency, continuing until 2y, timing, food types) |
| BF/CF, breastfeeding and complementary feeding; MM, micromodule; PDS, public distribution system; RM, revision module; SHG, self-help group | |

A quasi-experimental evaluation of a nutrition behavior change intervention delivered through women’s self-help groups in rural India: impacts on maternal and young child diets, anthropometry and intermediate outcomes - Scott et al. – Online Supplementary Material

**Supplemental Table 2. Indicator definitions**

| **Indicator** | **Definition** |
| --- | --- |
| **Child’s diet** | |
| Child minimum diet diversity (MDD) | Percentage of children 6-23 months of age who received foods from at least 5 out of 8 food groups during the previous day. The 8 food groups include: Breastmilk; Grains, roots, tubers and plantains; Pulses (beans, peas, lentils), nuts and seeds; Dairy products (milk, infant formula, yogurt, cheese); Flesh foods (meat, fish, poultry, organ meats); Eggs; Vitamin-A rich fruits and vegetables; Other fruits and vegetables |
| Child minimum meal frequency (MMF) | Percentage of children 6-23 months of age who received solid, semi-solid, or soft foods (but also including milk feeds for non-breastfed children) the minimum number of times or more during the previous day. Minimum number of times is defined as 2 feedings of solid, semi-solid, or soft foods for breastfed infants 6-8 months, 3 feedings of solid, semi-solid, or soft foods for breastfed children 9-23 months and 4 feedings of solid, semi-solid, or soft foods or milk feeds for non-breastfed children 6-23 months of which at least 1 of the 4 feeds must be a solid, semi-solid or soft feed. |
| Child minimum acceptable diet (MAD) | Proportion of index children 6-23.9 mo who had both MDD and MMF on the previous day |
| **Child’s anthropometry** | |
| Child height/length-for-age z score (HAZ) | Continuous indicator according to World Health Organization age and sex specific cutoffs |
| Child weight-for-age z score (WAZ) | Continuous indicator according to World Health Organization age and sex specific cutoffs |
| Child weight-for-height z score (WHZ) | Continuous indicator according to World Health Organization age and sex specific cutoffs |
| Child stunting | Proportion of index children with HAZ < -2 |
| Child underweight | Proportion of index children with WAZ < -2 |
| Child wasting | Proportion of index children with WHZ < -2 |
| **Woman anthropometry** | |
| Woman BMI | Respondent woman’s weight in kg divided by her height in meters squared |
| Woman underweight | Proportion of respondent women with BMI < 18.5 kg/m^2^ |
| **Woman’s diet** | |
| Minimum diet diversity (MDD-W) | Proportion of respondent women who consumed at least 5 of 10 food groups on the previous day; indicator follows the FANTA guidelines [1]. The five food groups include Staples (cereals, tubers); Pulses; Nuts and seeds; Dairy; Flesh foods (meat, poultry, fish); Eggs; Green leafy vegetables; Vit A rich fruit and vegetables; Other vegetables; Other fruits |
| Consumption of animal source foods | Proportion of respondent women who consumed meat, poultry, fish, eggs or dairy on the previous day |
| **Woman’s knowledge** | |
| Knowledge indicators are calculated using questions asked to women based on BCC content in different sub-sections. Each question is given a score of 1 based on correct response(s). Scores on each subsection are then converted to a scale of 0 -100 to interpret in percentage terms. | |
| Knowledge score on child feeding | Includes knowledge sub-scores: BF initiated within 1hr of birth; colostrum feeding; ensuring enough breastmilk to baby; giving water to child under 6 months; introducing animal source foods to child timely (6-8 mos.); prepare food for ease of consuming by young child (at least 2 correct); number of months for excl. breastfeeding (6mos); breastfeeding until correct age (24mos); age of initiation of complementary feeding (6mos) |
| Knowledge score on child health, hygiene and sanitation | Includes knowledge sub-scores: treatment for child having diarrhoea (at least 2 correct); ways to protect a child from getting worms; when mother of a young child should wash hands (at least 3 correct) |
| Knowledge score on maternal health and nutrition | Includes knowledge sub-scores: problems faced by women married too early; number of ANC check-ups for a pregnant woman; recognising person with anaemia; prevention measures for anaemia |
| Knowledge score on dietary diversity | Includes knowledge sub-scores: food items rich in iron; food items that provide body energy; food items that help body grow; food items that protect from illnesses; food items rich in Vitamin-A |
| Overall knowledge score | Overall score includes knowledge scores on: Knowledge score on child feeding; child health, hygiene and sanitation; maternal health and nutrition; dietary diversity |
| **Woman’s antenatal care** | |
| ANC quality: total | Scaled (0-1) sum score of all clinical checks conducted and advice given during ANC during pregnancy with index child, 0-12 (bp measured, urine sample, blood sample, abdomen checked, told expected delivery date, advised to deliver in health facility, advised about IFA, advised about calcium, advised about side effects of IFA, advised about side effects of calcium, advised about early initiation of BF, weighed) |
| ANC quality: clinical | Scaled (0-1) sum score of clinical checks conducted during ANC during pregnancy with index child, 0-5 (bp measured, urine sample, blood sample, abdomen checked, weighed) |
| ANC quality: advice | Scaled (0-1) sum score of advice given during ANC during pregnancy with index child, 0-7 (told expected delivery date, advised to deliver in health facility, advised about IFA, advised about calcium, advised about side effects of IFA, advised about side effects of calcium, advised about early initiation of BF) |
| **Woman’s decision making and gender attitude** | |
| Decision making – women’s diet | Adequate if respondent is the sole decision maker or contributes at least to a medium extent to input into decisions to: foods to prepare; foods to eat |
| Decision making – women’s health and diet during pregnancy | Adequate if respondent is the sole decision maker or contributes at least to a medium extent to input into decisions to: consult doctor; work; rest; eat eggs; eat milk or milk products; eat meat |
| Decision making – child’s diet | Adequate if respondent is the sole decision maker or contributes at least to a medium extent to input into decisions to feeding child: eggs; milk and milk products; meat |
| Decision making – breastfeeding and complementary feeding | Adequate if respondent is the sole decision maker or contributes at least to a medium extent to input into decisions to child below 2 years of age: how to feed when sick; whether to breastfeed; when to stop breastfeeding; when to start complementary feeding to IC |
| **Household** |  |
| HFIAS score | Standard HFIAS indicator ranging from 0 to 27, with higher value indicating higher food insecurity in the past 4 weeks |
| Food secure | Proportion of households that experiences none of the food insecurity (access) conditions, or just experiences  worry, but rarely. |
| Household production diversity | Scaled (0-10) sum score of (0-34) of the food items – staples, oilseeds/pulses, vegetables, fruits and livestock/poultry/fish/meat/eggs – grown/produced by HH in last 12 months |
| Progressive attitudes score | Scaled (0-1) score of attitudes toward gender issues (husband should help in house, husband should not let wife work outside home, woman has right to express own opinion, woman must accept husband beats her, better to send son to school than daughter); a higher score indicates more gender equal attitudes |
|  |  |

[1] <https://www.fantaproject.org/monitoring-and-evaluation/minimum-dietary-diversity-women-indicator-mddw>

| **Supplemental Table 3. Data collection and data quality checks** | |
| --- | --- |
| Survey firm responsibilities | Data collection was subcontracted to Oxford Policy Management (OPM), who were responsible for translation and programming of the survey instruments, enlisting and training the enumerators, and supervising data collection, data entry, and initial data cleaning. |
| Pre-testing survey tools | All the tools were pre-tested in control areas outside the evaluation primary sampling units. The pre-test was conducted jointly by OPM and IFPRI in October 2019. Revisions were made to the tools following this pre-test, and following feedback received from the PRADAN and PHRS team. The translation of the final tools was shared with the partners, and multiple revisions to language used were made at the request of the local PRADAN team and post discussions with the OPM field trainers. |
| CAPI programming | OPM collected data using Computer Assisted Personal Interviewing (CAPI) on tablets built through CSPro data entry platform. While CAPI is demonstrated to have clear benefits in terms of data quality, its success depends substantially on the effort spent programming, piloting and testing the application. Programming of the CAPI was done with several inbuilt checks-unique identification dropdowns, auto-populated baseline data, module-wise instructions, automated skips, color coding, consistency and range checks, digitally recorded date, time and GPS coordinates, etc.- to avoid enumerator errors, administer correct questions, monitor interview length and tracking locations. Iterative beta and gamma testing were conducted to the debug the CAPI instruments before and during the training for enumerators and supervisors. |
| Enumerator training | Training for enumerators and supervisors was conducted between November 25th to December 15th, 2020 at Jabalpur, Madhya Pradesh. The training was led by OPM India’s Survey Training and Quality Assurance specialist and attended by researchers from OPM and IFPRI. Over the course of the training, hands on practice sessions using CAPI on tablets, sessions in the field, role-playing and debrief sessions were conducted. Apart from detailed training on the protocols and tools, the training also covered themes such as objectivity, research ethics and data transmission. A fieldwork guidance manual covering fieldwork procedure, a debriefing note describing each tool and a document containing specific terms in local languages, were developed for the enumerators and fieldwork supervisors and shared during the training. |
| Field team structure | Given the sample size and timeline, OPM recruited a team of 35 female members. Different teams of enumerators and supervisors simultaneously conducted fieldwork for the Hindi-speaking states (MP, Chhattisgarh, Jharkhand), Odisha and West Bengal. Each primary sampling unit was covered in approximately 2-3 days depending on its size. At the ground level, each team consisted of three female enumerators, one supervisor and two health investigators. Overall, enumerators were responsible for data collection, data transfer and any other specific tasks agreed during the training. Each supervisor was responsible for their field teams, back checks, monitoring and any other task agreed during the training. Coordinators monitored field work movement, managed day to day field logistics and reported to the field director. |
| Data quality checks | Fieldwork movement was supervised by field visits, monitoring of field logistics on a daily basis and data quality auditing on real-time survey data. IFPRI research team and OPM’s data management team regularly monitored the real-time data shared by field teams using STATA 16 to ensure that there were no inconsistencies in data though error check protocols provided. The error check results were used to debrief enumerators daily and shared with IFPRI on a regular basis through documented field progress and error frequency reports. Further, spot and back checks were implemented in the field by supervisors, coordinators and OPM staff to monitor the enumerator’s performance, ensure that the survey protocol is adhered to and facilitate midstream corrections in real time. Spot and back checks were conducted on 10 percent of the households. For back checks, supervisors returned to selected households after the field teams had completed data collection, to cross-check the information gathered during the interview. Revisits were conducted to rectify errors that were detected during concurrent data quality monitoring while the survey was in progress. Progress updates were shared with IFPRI on a bi-weekly basis, detailing survey progress, errors or issues encountered in the field and updates from teams debriefs. |

**Supplemental Table 4. Propensity score regression estimation based on logit model of program participation at baseline**

| **Covariate [N=1337]** | ***Estimate ± SE*** | ***p value*** |
| --- | --- | --- |
| **Mother** |  |  |
| Age, years | 0.08 ± 0.11 | 0.47 |
| Age, years (squared) | -0.00 ± 0.00 | 0.57 |
| Completed education, years | 0.01 ± 0.02 | 0.68 |
| Age at marriage | -0.01 ± 0.03 | 0.67 |
| Father-in-law present, % | -0.02 ± 0.17 | 0.89 |
| Mother-in-law present, % | -0.03 ± 0.16 | 0.85 |
| **Household** |  |  |
| HH size, no. of members | -0.04 ± 0.04 | 0.39 |
| HH head caste: Scheduled Caste, % | -0.38 ± 0.37 | 0.31 |
| HH head caste: Scheduled Tribe, % | -0.81 ± 0.35 | 0.02** |
| HH head caste: OBC, % | -0.38 ± 0.36 | 0.29 |
| Uses improved drinking water source, % | -0.30 ± 0.13 | 0.02** |
| Any HH member has bank account, % | -0.08 ± 0.27 | 0.77 |
| Ratio of male to female HH members | -0.04 ± 0.07 | 0.60 |
| Dependency ratio (<15y + >55y : 16-55y) | 0.06 ± 0.11 | 0.56 |
| Has electricity, % | 0.18 ± 0.16 | 0.26 |
| Main roof made of improved material, % | -0.31 ± 0.16 | 0.06** |
| Main floor made of improved material, % | 0.22 ± 0.19 | 0.26 |
| Main exterior wall made of improved material, % | -0.09 ± 0.16 | 0.59 |
| Highest completed education for HH females, years | -0.01 ± 0.00 | 0.26 |
| **Village** |  |  |
| Women's average years of education per village | 0.21 ± 0.04 | 0.00*** |
| Wealth index at village level, leave-out mean | -0.33 ± 0.08 | 0.00*** |
| **Child** |  |  |
| Age, months | -0.01 ± 0.01 | 0.36 |
| Constant | -0.90 ± 1.54 | 0.56 |

Legend: *** p<0.01; ** p<0.05; * p<0.10

**Supplemental Table 5. Kernel weighted means of the covariates at baseline**

| **Weighted Variable(s)** | | **NI [N=833]** | **STD [N=766]** | | **Difference** | **p-value** |  |  |
| --- | --- | --- | --- | --- | --- | --- | --- | --- |
| **Mother** | |  | | |  | | | |
| Age, years | | 25.65 | 25.73 | | -0.09 | 0.72 |  |  |
| Age, years (squared) | | 678.80 | 683.20 | | -4.41 | 0.75 |  |  |
| Completed education, years | | 5.10 | 4.88 | | 0.23 | 0.34 |  |  |
| Age at marriage | | 18.31 | 18.27 | | 0.05 | 0.72 |  |  |
| Father-in-law present, % | | 0.29 | 0.27 | | 0.02 | 0.37 |  |  |
| Mother-in-law present, % | | 0.38 | 0.37 | | 0.01 | 0.80 |  |  |
| **Household** | |  | | |  | | | |
| HH size, no. of members | | 5.42 | 5.42 | | -0.00 | 0.98 |  |  |
| HH head caste: Scheduled Caste, % | | 0.16 | 0.15 | | 0.00 | 0.81 |  |  |
| HH head caste: Scheduled Tribe, % | | 0.52 | 0.52 | | -0.01 | 0.76 |  |  |
| HH head caste: OBC, % | | 0.29 | 0.28 | | 0.01 | 0.74 |  |  |
| Uses improved drinking water source, % | | 0.69 | 0.71 | | -0.02 | 0.34 |  |  |
| Any HH member has bank account, % | | 0.97 | 0.95 | | 0.00 | 0.79 |  |  |
| Ratio of male to female HH members | | 1.14 | 1.14 | | -0.00 | 0.99 |  |  |
| Dependency ratio (<15y + >55y : 16-55y) | | 1.11 | 1.14 | | -0.03 | 0.49 |  |  |
| Has electricity, % | | 0.84 | 0.84 | | 0.00 | 0.95 |  |  |
| Main roof made of improved material, % | | 0.15 | 0.15 | | 0.00 | 0.91 |  |  |
| Main floor made of improved material, % | | 0.24 | 0.24 | | -0.01 | 0.81 |  |  |
| Main exterior wall made of improved material, % | | 0.36 | 0.36 | | -0.00 | 0.94 |  |  |
| Highest completed education for HH females, years | | 8.44 | 7.90 | | 0.53 | 0.44 |  |  |
| **Village** | |  | | |  | | | |
| Women's average years of education per village | | 5.031 | 4.87 | | 0.16 | 0.17 |  |  |
| Wealth index at village level, leave-out mean | | -0.06 | -0.11 | | 0.06 | 0.28 |  |  |
| **Child** | |  | | |  | | | |
| Age, months | | 15.26 | 15.33 | | -0.08 | 0.78 |  |  |

| **Supplemental Table 6. Comparison of exposure, knowledge, and diet indicators for nutrition intensive and standard groups in 2015 and 2017 from WINGS panel sample** | | | | |
| --- | --- | --- | --- | --- |
|  | **2015** | | **2017** | |
|  | **NI**  **[N=757]** | **STD [N=696]** | **NI  [N=757]** | **STD [N=696]** |
| **Exposure: heard story about…** |  |  |  |  |
| Character: Soni | NA | NA | 11 | 2 |
| Character: Madhu | NA | NA | 10 | 1 |
| Character: Silvanti | NA | NA | 7 | 2 |
| **Woman’s knowledge indicators** |  |  |  |  |
| Heard of iron deficiency anemia | 35 | 34 | 69 | 66 |
| Don’t know any foods rich in vitamin A | 53 | 52 | 50 | 52 |
| Don’t know why nutrition is important for pregnant women | 38 | 40 | 8 | 11 |
| Don’t know when a caretaker should wash hands | 13 | 12 | 1 | 1 |
| Don’t know how to protect child from worms | 47 | 48 | 42 | 41 |
| Say that breastfeeding should be initiated within 1 hour after birth | 73 | 72 | 83 | 85 |
| Say that colostrum should be given to child | 63 | 63 | 69 | 69 |
| **Women’s diet and nutritional status** |  |  |  |  |
| Achieved minimum dietary diversity | 9 | 10 | 15 | 15 |
| Underweight | 46 | 45 | 42 | 38 |
| All numbers are percentages  NA = not applicable, exposure data was not collected in 2015 | | | | |
